# MRSA Septic Thrombophlebitis and Pulmonary Embolism: A Case Report Highlighting the Tight Bond With Infective Endocarditis

**DOI:** 10.1002/ccr3.73077

**Published:** 2026-07-06

**Authors:** Santiago Saenz‐Ancira, Javier Higuera‐Ornelas, Lorenzo Olivero, Ermin Tale, Pawel Borkowski, Suraj Adhikari

**Affiliations:** ^1^ Department of Internal Medicine Jacobi Medical Center/Albert Einstein College of Medicine New York City New York USA; ^2^ Department of Human Genetics McGill University Montréal Quebec Canada

**Keywords:** bacteremia, infective endocarditis, methicillin‐resistant *Staphylococcus aureus*, septic pulmonary embolism, septic thrombophlebitis

## Abstract

Septic pulmonary embolism in adults is usually associated with right‐sided endocarditis. We report a 27‐year‐old male with 14 days of fever, dyspnea, and hemoptysis. CT chest showed bilateral peripheral cavitating nodules consistent with septic pulmonary emboli. Blood cultures yielded MRSA; transesophageal echocardiography showed no vegetations. CT abdomen‐pelvis identified a thrombus in the inguinal venous system compatible with septic thrombophlebitis. Management included IV antibiotics and anticoagulation. His case highlights the close interplay between septic thrombophlebitis and infective endocarditis. As well as the utility of prompt extra‐thoracic imaging when endocarditis work up is negative. Central discussion points include the devastating consequences of persistent bacteremia, urging the clinician to take action and the controversial use of anticoagulation for septic pulmonary embolism, which in this case was completely beneficial.

## Key Clinical Message

A key differential diagnosis for endocarditis is septic thrombophlebitis. This entity is not necessarily related to the use of intravascular catheters. Persistent bacteremia is associated with a substantial increase in mortality and should urge intervention. The use of anticoagulation must be individualized given the lack of recommendations and large studies.

## 1 Background

Septic thrombophlebitis often occurs in association with an intravenous catheter, internal jugular vein thrombosis after oropharyngeal infection (Lemierre syndrome) or pylephlebitis arising from intra‐abdominal infections, though contiguous spread from adjacent nonvascular foci also occurs [1, 2]. These endovascular infections can seed septic pulmonary emboli (SPE), which are frequently composed of *Staphylococcus aureus* (including MRSA) [3]. In adults, particularly those presenting with *Staphylococcus aureus* bacteremia, right‐sided infective endocarditis (IE) is a key differential; although transesophageal echocardiography (TEE) is highly sensitive, early studies can be negative, and guidelines advise repeating TEE when the clinical suspicion persists [4]. Diagnosis rests on microbiology plus imaging; persistent bacteremia despite appropriate therapy should prompt evaluation for an endovascular source. Peripheral venous sources outside the jugular or portal systems (e.g., inguinal or iliac veins) are less commonly reported causes of SPE. We report MRSA SPE arising from inguinal venous septic thrombophlebitis with a negative TEE, underscoring the need to seek non‐cardiac peripheral sources when IE work‐up is unrevealing [3].

## 2 Description of the Case

A 27‐year‐old male presented to the Emergency Department with a 14‐day course of fever, dyspnea and hemoptysis. Vitals at arrival revealed tachycardia 137 bpm and fever 39.3°C. Physical exam notable for multiple draining purulent wounds of the lower back (Figure 2B) and bilateral pulmonary inspiratory rales. Laboratory tests revealed leukocytosis (33.7 × 10^9^/L, with a left shift), a lactate level of 2.4 mmol/L, and an INR of 1.9. Contrast‐enhanced chest CT demonstrated a 4.0 × 3.8 × 4.2 cm thick‐walled right upper‐lobe cavity, as well as multiple bilateral cavitating pulmonary nodules (Figure 1). Two sets of blood cultures and a nasal swab were positive for MRSA, and the patient was started on antibiotic treatment consisting of intravenous vancomycin with trough‐guided dosing. The patient also underwent incision and drainage of the wounds in the lower back.

**FIGURE 1:**
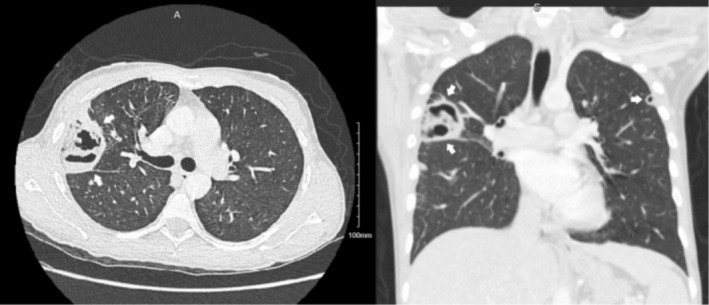
CT pulmonary angiography showing bilateral peripheral cavitating nodules (white arrows) and a thick‐walled right upper‐lobe cavity (4.0 × 3.8 × 4.2 cm) with tree‐in‐bud nodularity.

Daily blood cultures were monitored; however, the patient remained bacteremic until day 8 of admission. A TEE on hospital day 6 was negative for any signs of endocarditis. After day 5 of bacteremia, a decision was made to discontinue vancomycin and begin treatment with daptomycin and ceftaroline. After ruling out endocarditis and having persistent bacteremia, source control of the infection became a concern. Therefore, given the initial soft tissue abscess identified on the physical exam, the patient underwent a CT abdomen–pelvis with contrast to evaluate for additional foci of infection. The imaging revealed inflammatory soft tissue changes in the lower back and a thrombus in the right common iliac/inguinal venous system consistent with septic thrombophlebitis (Figure 2A). The course was complicated with acute hypoxemic respiratory failure; chest radiograph revealed left‐sided spontaneous pneumothorax secondary to the rupture of a cavitary lesion (Figure 3A); a chest tube was placed with resolution (Figure 3B).

**FIGURE 2:**
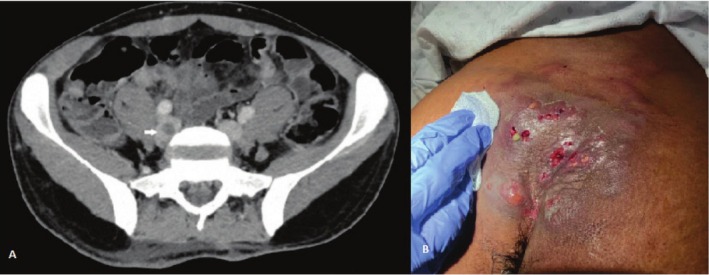
Contrast CT abdomen–pelvis showing right common iliac thrombus (white arrow) (A). Lower‐back draining purulent wounds (B).

**FIGURE 3:**
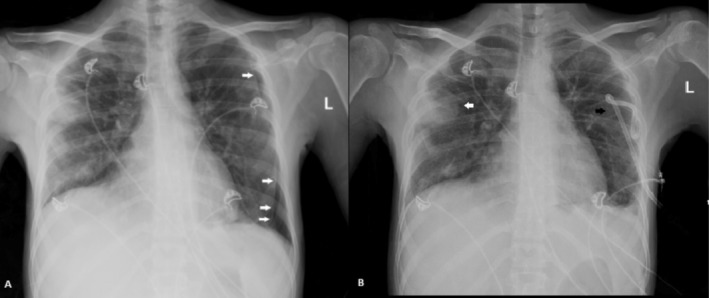
Anteroposterior chest radiograph showing left pneumothorax (white arrows) (A) and post–chest‐tube placement (black arrow) with persistent right upper‐lobe cavity (white arrow) (B).

Despite the placement of a chest tube and resolution of pneumothorax, the patient was persistently hypoxemic. He developed right testicular pain and swelling; CT and testicular ultrasound (Figure 4) confirmed the presence of a new‐onset varicocele. The patient was started on anticoagulation with enoxaparin for treatment of septic pulmonary embolism and septic thrombophlebitis. The blood cultures cleared on day 8 of admission, his clinical status continued to improve, and he was switched to apixaban. He was discharged on levofloxacin and rifampin, based on culture sensitivities, to complete a 5‐week course of antibiotics. Follow‐up imaging 8 weeks later revealed resolution of the pulmonary cavitations.

**FIGURE 4:**
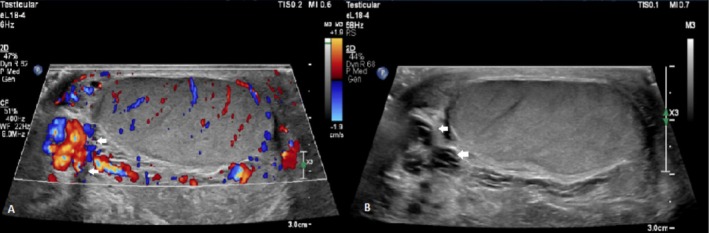
Testicular ultrasound demonstrating dilated pampiniform veins (varicocele): Color Doppler (A) and grayscale (B) (white arrows).

## 3 Discussion

Septic thrombophlebitis and infective endocarditis (IE) share many pathophysiological features, for example, fever, bacteremia, embolic phenomena, overlapping pathogens, and shared risk factors including intravenous drug use and the presence of indwelling intravascular devices [1, 2, 3, 4].

Our patient's chest CT presented with multiple bilateral peripheral cavitation nodules, a pattern highly compatible with SPE in the setting of bacteremia when compared to alternative causes of cavitary lung disease [3, 5]. *Staphylococcus aureus* bacteremia raises concern for right‐sided IE. Guideline‐based practice is to repeat echocardiography only when pretest probability remains high; in this case, identification of an extracardiac venous source lowered our suspicion of IE being the driver of the pulmonary lesions [4]. The series of events and findings, lower‐back soft tissue abscess with contiguous spread to the inguinal/common iliac venous system, culminating in septic thrombophlebitis and embolization, fits recognized non‐cardiac sources of SPE and explains the pulmonary pattern observed on imaging [1, 3]. This pathway contrasts with the classic jugular thrombophlebitis of Lemierre syndrome and the portal venous involvement of pylephlebitis, underscoring that peripheral venous systems outside the jugular or portal systems can seed SPE and should be evaluated when IE work‐up is unrevealing [1, 2, 3].

Our patient was started on vancomycin for the treatment of MRSA bacteremia. However, after 5 days of persistent bacteremia, we decided to switch the patient to ceftaroline and daptomycin due to concern for reduced vancomycin efficacy in the setting of persistent bacteremia and the known association between prolonged bacteremia and adverse outcomes [6, 7]. Persistent *S. aureus* bacteremia > 48 h carries a higher 90‐day mortality (39% vs. 22% when bacteremia clears within 24 h); each additional day confers 16% increased risk of death. Accordingly, early regimen optimization and prompt source control are critical in MRSA bacteremia [7, 8].

The role of anticoagulation in septic pulmonary embolism remains unclear in the medical literature [9, 10]. The efficacy and safety remain an unexplored area, and the decision to start is not evidence‐based but common in practice [9]. The decision, therefore, must be individualized, taking into consideration the risks and benefits applied to the patient's particular case [9, 10]. Regarding our patient, we were concerned about a high thrombotic burden in the pulmonary arteries and iliac venous system leading to his clinical deterioration; therefore, we decided to anticoagulate. The rationale behind the potential benefit is to counteract the prothrombotic state induced by bacteremia [10]. However, it must be closely monitored, given that patients with sepsis are also at risk for disseminated intravascular coagulation, requiring replenishment of coagulation factors such as fibrinogen [10]. For our patient, his fibrinogen levels were continuously above 500, making the likelihood of overt disseminated intravascular coagulation and bleeding risk less concerning, even with the INR being elevated.

MRSA bacteremia is a clinical entity with high mortality. Its association with SPE can lead to severe complications and a worse prognosis. Prompt recognition and early therapy are paramount to ensure survival.

## Author Contributions

**Santiago Saenz‐Ancira:** investigation, project administration, writing – original draft, writing – review and editing. **Javier Higuera‐Ornelas:** writing – original draft, writing – review and editing. **Lorenzo Olivero:** investigation, writing – review and editing. **Ermin Tale:** writing – review and editing. **Suraj Adhikari:** writing – review and editing. **Pawel Borkowski:** validation, writing – review and editing.

## Funding

The authors have nothing to report.

## Ethics Statement

Verbal and signed consent from the patient was obtained. No approval was needed from the ethics committee.

## Consent

Written informed consent was obtained from the patient for publication of this case report and any accompanying images. A copy of the written consent is available for review by the Editor‐in‐Chief of this journal.

## Conflicts of Interest

The authors declare no conflicts of interest.

## Data Availability

The authors have nothing to report.
